# Supplementary material for: Differences in the inflammatory proteome of East African and Western European adults and associations with environmental and dietary factors
Source: eLife. 2023 Aug 9;12:e82297. doi: 10.7554/eLife.82297 (PMC10473835; doi:10.7554/eLife.82297)
Supplement: Supplementary file 1. — P- value unadjusted. [file elife-82297-supp1.docx]

| Loci | Cytokine | SNPs | Chr | Base pair | Protein | P-value | Direction | A1 | A2 |
| --- | --- | --- | --- | --- | --- | --- | --- | --- | --- |
| 1 | IL-1β | rs12169244 | 22 | 47,558,596 | TNFSF14 | 0.000928 | Increase | G | A |
| 2 | IL-1β | rs11829172 | 12 | 98,579,287 | CDCP1 | 0.021669 | Decrease | T | C |
| 3 | IFN-γ | rs4474665 | 16 | 17,498,563 | CDCP1 | 0.018818 | Increase | T | C |
| 4 | IL-6 | rs74115411 | 1 | 53,331,776 | ENRAGE | 0.000936 | Increase | T | A |
| 5 | IL-1β | rs9563018 | 13 | 51,656,046 | CCL25 | 0.012443 | Increase | A | G |
